# Editorial: Experiences and advances in endocrinology point-of-care ultrasound (POCUS)

**DOI:** 10.3389/fendo.2022.1094024

**Published:** 2023-01-23

**Authors:** Gustavo Bittenourt Camilo, Fikri Abu-Zidan, Abhilash Koratala

**Affiliations:** ^1^Department of Anatomy, Juiz de Fora Federal University, Juiz de Fora, Brazil; ^2^The Research Office, College of Medicine and Health Sciences, United Arab Emirates University, AlAin, United Arab Emirates; ^3^College of Health Sciences, University of Wisconsin–Milwaukee, Milwaukee, WI, United States

**Keywords:** POCUS, ultrasound, endocrinology, radioablation, imaging technics

Ultrasound is one of the most important imaging techniques in medicine and has endless advantages including portability, non-invasiveness, low cost, absence of radiation or contrast, real-time imaging, ease of use, and bedside assessment, in addition to facilitating decisions in medical practice (1–3). This method provides the physician with greater skill in caring for patients and improves the information collected upon physical examination (4, 5).

Advances in technology associated with the growth of medical ultrasound education in undergraduate studies have facilitated the integration of point-of-care ultrasound (POCUS) not only in radiology and cardiology but also into a wider variety of fields such as anesthesia and emergency medicine (6, 7). In endocrinology, the use of ultrasonography is also widely used, and the evaluation of the thyroid should be highlighted (4, 8).

It is important to emphasize that this imaging technique is also being used to guide many procedures, biopsies, and surgeries (9).

The objective of the Research Topic “*Experiences and advances in endocrinology point-of-care ultrasound (POCUS)*” was to gather original research articles and review illustrations of the recent advances concerning the roles of ultrasound and its support in clinical practice especially in endocrinology. This Research Topic consists of five original articles.

Two original articles deal with the theme interventional ultrasound to guide procedures in thyroid. Minimally invasive measures are applied in nodules and tumors like laser ablation, ethanol ablation, and radiofrequency ablation. Both articles highlighted the importance of diagnosing thyroid nodules and predicting the differentiation between benign and malignant lesions and is never too much to emphasize that ultrasound is the first-line imaging examination for the malignancy risk assessment of thyroid nodules (1). Yong-ping et al. investigated the efficacy and safety of ultrasound-guided percutaneous laser ablation for treating recurrent papillary thyroid cancer nodules. They showed that the ultrasound-guided laser ablation is minimally invasive and common for patients with thyroid nodules and is a clinically effective, repeatable, and efficient outpatient treatment that is well-tolerated and is associated with a low risk of major complications. They concluded that the procedure appears to be effective and safe in treating unifocal recurrent nodules in selected patients who are ineligible for surgery, which is suitable for clinical application and promotion. Lin et al. investigated the efficacy and safety of radiofrequency ablation among cases attacked by large benign solid thyroid nodules, mainly focusing on volume reduction, complication rate, and thyroid function. They concluded that radiofrequency ablation is an efficient measure to treat large benign solid thyroid nodules, showing minimal invasion and reaching complete ablation to solve compression symptoms. Single or sequential regimen might ablate large nodules with great safety and assurance. The importance of ultrasound was highlighted in the different phases of the process from diagnosis to guide the procedure, assessment of the therapeutic response, and follow-up of patients.

The third article deals with the efficacy and safety of ultrasound-guided microwave ablation in the treatment of primary hyperparathyroidism (PHPT). Ni et al. aimed to investigate the changes in PTH, serum calcium, and volume of ablated lesions in hyperparathyroidism patients, and evaluate skeletal and renal functions to further evaluate the efficacy and safety of ultrasound-guided procedure. They verified that the procedure is a reasonable option in the treatment of PHPT, especially asymptomatic patients with potential target organ damage or a mild condition. They concluded that ultrasound-guided ablation effectively decreases levels of abnormal bone turnover markers and thus improve bone health of these patients. It is important to highlight that the evaluation of the parathyroid glands is even more difficult due to the small size of these anatomical structures, and ultrasound is fundamental in the procedure.

The fourth article deals with the predictors of central cervical lymph node metastasis for papillary thyroid carcinoma and establishes a prediction model to guide the operation of these patients. As they pointed out in their article, “US features, including spotty microcalcifications and aspect ratio, are vital information for differentiating malignant nodules from benign nodules.” Gao et al. accentuated the ultrasound features like nodule size, boundary, shape, internal echo, aspect ratio, capsular contact, microcalcifications, blood flow, and the procedure guided by ultrasound. They identified the following three significant independent predictors for central cervical lymph node metastasis in papillary thyroid carcinoma: tumor size (measured by ultrasound), US features (microcalcifications), and thyroglobulin antibodies (being positive).

Finally, the last original article brings innovation, It is an eveolving theme which deals with dynamic artificial intelligence (AI) ultrasound auxiliary diagnosis system. Wang et al. investigated the value of dynamic AI in differentiating benign and malignant thyroid nodules and guiding treatment strategies. They discussed that diagnostic results are affected by several factors such as operator's experience and skills, ultrasound equipment, and thyroid basic lesions, and concluded that the dynamic AI examination has high diagnostic value for benign and malignant thyroid nodules, which can effectively assist surgeons in planning individualized diagnosis and treatment strategies for patients.

In summary, the articles presented in the Research Topic “*Experiences and Advances in Endocrinology Point-of-Care Ultrasound (POCUS)*” provide a useful source of information concerning the role of ultrasound in endocrinology from diagnosis to guide procedures, assessment of the therapeutic response, and follow-up of patients.

## Author contributions

All authors listed have made a substantial, direct, and intellectual contribution to the work, and approved it for publication.
